# Maintenance treatment with esomeprazole following initial relief of non-steroidal anti-inflammatory drug-associated upper gastrointestinal symptoms: the NASA2 and SPACE2 studies

**DOI:** 10.1186/ar2124

**Published:** 2007-02-09

**Authors:** Christopher J Hawkey, Nicholas J Talley, James M Scheiman, Roger H Jones, Göran Långström, Jorgen Næsdal, Neville D Yeomans, the NASA/SPACE author group

**Affiliations:** 1 Institute of Clinical Research, Trials Unit, Wolfson Digestive Diseases Centre, University Hospital, Derby Road, Nottingham, NG7 2UH, UK; 2 Mayo Clinic College of Medicine, 200 First Street SW, Rochester, MN 55905, USA; 3 Division of Gastroenterology, Department of Internal Medicine, University of Michigan Medical Center, 3912 Taubman Center, Ann Arbor, MI 48109-0362, USA; 4 GKT Department of General Practice, King's College, 5 Lambeth Walk, London, SE11 6SP, UK; 5 AstraZeneca R&D, Karragatan 5, Pepparedsleden 1, Mölndal 431 83, Sweden; 6 Medical School, University of Western Sydney, Locked Bag 1797, Penrith South DC, NSW 1797, Australia

## Abstract

Non-steroidal anti-inflammatory drugs (NSAIDs), including selective cyclo-oxygenase-2 (COX-2) inhibitors, cause upper gastrointestinal (GI) symptoms that are relieved by treatment with esomeprazole. We assessed esomeprazole for maintaining long-term relief of such symptoms. Six hundred and ten patients with a chronic condition requiring anti-inflammatory therapy who achieved relief of NSAID-associated symptoms of pain, discomfort, or burning in the upper abdomen during two previous studies were enrolled and randomly assigned into two identical, multicentre, parallel-group, placebo-controlled studies of esomeprazole 20 mg or 40 mg treatment (NASA2 [Nexium Anti-inflammatory Symptom Amelioration] and SPACE2 [Symptom Prevention by Acid Control with Esomeprazole] studies; ClinicalTrials.gov identifiers NCT00241514 and NCT00241553, respectively) performed at various rheumatology, gastroenterology, and primary care clinics. Four hundred and twenty-six patients completed the 6-month treatment period. The primary measure was the proportion of patients with relapse of upper GI symptoms, recorded in daily diary cards, after 6 months. Relapse was defined as moderate-to-severe upper GI symptoms (a score of more than or equal to 3 on a 7-grade scale) for 3 days or more in any 7-day period. Esomeprazole was significantly more effective than placebo in maintaining relief of upper GI symptoms throughout 6 months of treatment. Life-table estimates (95% confidence intervals) of the proportion of patients with relapse at 6 months (pooled population) were placebo, 39.1% (32.2% to 46.0%); esomeprazole 20 mg, 29.3% (22.3% to 36.2%) (*p *= 0.006 versus placebo); and esomeprazole 40 mg, 26.1% (19.4% to 32.9%) (*p *= 0.001 versus placebo). Patients on either non-selective NSAIDs or selective COX-2 inhibitors appeared to benefit. The frequency of adverse events was similar in the three groups. Esomeprazole maintains relief of NSAID-associated upper GI symptoms in patients taking continuous NSAIDs, including selective COX-2 inhibitors.

## Introduction

Continuous users of non-steroidal anti-inflammatory drugs (NSAIDs) often experience treatment-associated upper gastrointestinal (GI) side effects, including upper abdominal pain and heartburn [1-3]. Although recently introduced drugs that selectively inhibit the cyclo-oxygenase-2 (COX-2) enzyme (selective COX-2 inhibitors) were expected to have better GI tolerability, it is clear that these drugs are also associated with a substantial level of drug-related symptomatic upper GI adverse events (AEs) requiring treatment [4,5].

Upper GI symptoms, together with the underlying inflammatory disease, lead to substantial reductions in health-related quality of life (HRQL) in patients taking long-term NSAID therapy [6,7] and often lead to dose reduction or discontinuation of NSAID treatment [3,8]. Treatments that relieve upper GI symptoms associated with NSAIDs may thus allow patients to continue therapy with these drugs. However, it is important that such additional treatments are themselves well tolerated and effective for long periods of time given that many patients with chronic inflammatory conditions require continuous NSAID therapy for many years.

In animal studies, NSAID-associated gastric mucosal damage has been shown to be highly pH-dependent [9]. Consequently, a combination of NSAID treatment and acid suppressive therapy may minimise the risk of NSAID toxicity and associated upper GI symptoms, and human studies have established a major role for proton pump inhibitors (PPIs) in this regard [10,11]. In addition, there are growing indications that PPIs are effective in relieving the upper GI symptoms associated with the use of NSAIDs, including selective COX-2 inhibitors [12].

We therefore conducted two pairs of placebo-controlled studies that evaluated the efficacy, tolerability, and safety of acid suppression with esomeprazole in the treatment and prevention of NSAID-associated upper GI symptoms. Each study consisted of an acute and a maintenance study. The acute 4-week symptom relief studies (Nexium Anti-inflammatory Symptom Amelioration [NASA1] and Symptom Prevention by Acid Control with Esomeprazole [SPACE1] studies) have shown that esomeprazole (20 and 40 mg) relieves upper GI symptoms in patients using NSAIDs, including selective COX-2 inhibitors [12]. In this paper, we report a comparison of esomeprazole 20 and 40 mg with placebo in the long-term (6 months) maintenance of relief of upper abdominal pain, discomfort, or burning in patients continuing to use NSAIDs, including selective COX-2 inhibitors.

## Materials and methods

### Study design

Two 6-month randomised, double-blind, parallel-group, placebo-controlled studies (NASA2 [SH-NEN-0002] and SPACE2 [SH-NEN-0004]) were conducted in 149 outpatient centres, including rheumatology, gastroenterology, and primary care, in Europe, USA, Canada, South Africa, and Australia. Signed informed consent was obtained from all patients prior to entry into the study. The study was approved by an independent ethics committee.

### Patients

Patients (male and female, at least 18 years old) who achieved relief of upper GI symptoms (pain, discomfort, or burning in the upper abdomen) in the NASA1 and SPACE1 studies, whether taking placebo, esomeprazole 20 mg, or esomeprazole 40 mg, were eligible for immediate inclusion in the subsequent 6-month maintenance studies. Relief during the acute studies was defined as having a diary assessment of 'none' or 'minimal' (scores of 0 or 1, respectively, on a 7-grade scale) on at least 5 of the final 7 days and no more than 'mild' (a score of 2) on no more than 2 days. Patients satisfying these criteria were re-randomised (in equal proportion in blocks of six according to a computer-generated randomisation list) upon entry to the 6-month studies to receive esomeprazole 20 mg, esomeprazole 40 mg, or placebo once daily for 6 months, in addition to their NSAID therapy.

All patients included had a chronic condition requiring continuous daily treatment with one or more oral NSAIDs (including selective COX-2 inhibitors and high-dose aspirin [>325 mg/day]) for at least 5 days in any given week for the duration of the study. Patients using low-dose aspirin (<325 mg/day) in conjunction with an NSAID or COX-2 inhibitor were allowed to participate in the study but were categorised as receiving non-selective NSAID therapy (reflecting the COX-1 inhibitory effects of this therapy) regardless of whether aspirin was used alone or in combination with a non-selective NSAID or a selective COX-2 inhibitor. For assessment of the effect of NSAID type on study variables, patients were included in the selective COX-2 inhibitor group only if they were taking a selective COX-2 inhibitor alone.

Exclusion criteria included pain, discomfort, or burning in the upper abdomen precipitated by exercise, relieved by defecation, or not associated with the use of NSAIDs; a history of symptomatic gastroesophageal reflux disease (GERD) not associated with the use of NSAIDs; a history of erosive esophagitis, gastric, or duodenal ulcer or of esophageal, gastric, or duodenal surgery; a need for concomitant therapy with drugs likely to affect the outcome of the study (stable treatment with disease-modifying anti-rheumatic drugs was permitted, as was limited glucocorticoid use).

### Assessments

The severity of NSAID-associated upper GI symptoms (pain, discomfort, or burning in the upper abdomen) was rated by the patient on daily diary cards on a 7-grade scale from 0 (none: no symptoms) to 6 (very severe: cannot be ignored and markedly limits daily activities and often requires rest). In addition, at baseline and after 1, 3, and 6 months, the severity of four other upper GI symptoms during the previous 7 days (heartburn, acid regurgitation, upper abdominal bloating, and nausea) was assessed by the investigator and graded from 0 (none: no symptoms) to 3 (severe: incapacitating with inability to perform normal activities).

Upper GI symptom severity and HRQL were also assessed using two validated patient-reported outcome instruments, the Gastrointestinal Symptom Rating Scale (GSRS) [13] and the Quality of Life in Reflux and Dyspepsia (QOLRAD) questionnaire [14,15]. Both instruments use 7-grade Likert scales and group related aspects into five dimensions: reflux, abdominal pain, indigestion, diarrhoea, and constipation (for the GSRS) and emotional distress, sleep disturbance, food/drink problems, vitality, and physical/social functioning (for the QOLRAD questionnaire). The QOLRAD questionnaire is a disease-specific instrument that was developed for patients with upper GI symptoms, including heartburn and dyspepsia. The GSRS is also a disease-specific instrument and was developed specifically to assess GI symptom severity. Mean change from baseline in the three QOLRAD dimensions of emotional distress, sleep disturbance, and food/drink problems was assessed, as was mean change from baseline in the three GSRS dimensions of reflux, abdominal pain, and indigestion. Compliance with NSAIDs and with the study drug was monitored by diary cards and by the investigator (counting unused tablets), respectively. AEs were monitored at 1, 3, and 6 months by the investigator.

### Statistical analyses

The primary variable was the proportion of patients with relapse of upper GI symptoms associated with the use of daily NSAIDs, including selective COX-2 inhibitors, after 6 months of treatment. Relapse was defined as moderate-to-severe upper GI symptoms (more than or equal to 3 on the 7-grade severity scale) on at least 3 days in any 7-day period. The proportions of patients with relapse of upper GI symptoms were analysed by life-table analysis (Kaplan-Meier estimates of time to first event) with differences between active treatment and placebo compared using the log-rank test. This analysis was performed for all patients and for those in the non-selective NSAID or selective COX-2 inhibitor subgroups. For analysis of the primary variable, the study populations were analysed separately. The two study populations were pooled for the assessment of the effect of NSAID type (non-selective versus selective COX-2 inhibitor).

The number needed to treat was calculated as the number of patients who need to be treated with esomeprazole 20 or 40 mg to avoid one case of upper GI symptom relapse, relative to placebo, during 6 months of treatment. Secondary variables included mean change in the three dimensions pre-specified as relevant to upper GI symptoms on each of the GSRS and QOLRAD questionnaires from baseline to last visit, analysed by analysis of covariance. The proportion of patients maintained free of investigator-assessed symptoms of heartburn, acid regurgitation, upper abdominal bloating, and nausea at 6 months was assessed *post hoc *with differences analysed by the Fisher exact test. A *post hoc *regression analysis using a Cox proportional hazards model was also performed to assess the influence of the following factors on time to relapse of pain, discomfort, or burning in the upper abdomen: age (less than 65 versus more than or equal to 65 years), gender (male versus female), *Helicobacter pylori *status (positive versus negative), selective COX-2 inhibitors (yes versus no), treatment during NASA1/SPACE1 (esomeprazole 40 mg, esomeprazole 20 mg, placebo), and treatment during NASA2/SPACE2 (esomeprazole 40 mg, esomeprazole 20 mg, placebo).

To determine the reliability of diary card measurements, the correlation coefficient for 7 days of diary card recordings was estimated using the GSRS abdominal pain domain. Pearson correlations between GSRS abdominal pain and mean of diary cards for the last 7 days were also assessed in terms of baseline values, last values, and change from baseline.

Each study was planned to include 300 patients in order to provide 90% power to detect a difference in upper GI symptom relapse rates of 19% for the esomeprazole groups and 42% for the placebo group at the significance level of 0.025 using the Fisher exact test. This was based on observed differences in relapse rates of respective variables between study drug and placebo in the ASTRONAUT (Acid Suppression Trial: Ranitidine versus Omeprazole for NSAID-Associated Ulcer Treatment) [11] and OMNIUM (Omeprazole versus Misoprostol for NSAID-Induced Ulcer Management) [10] studies.

## Results

**Figure 1:**
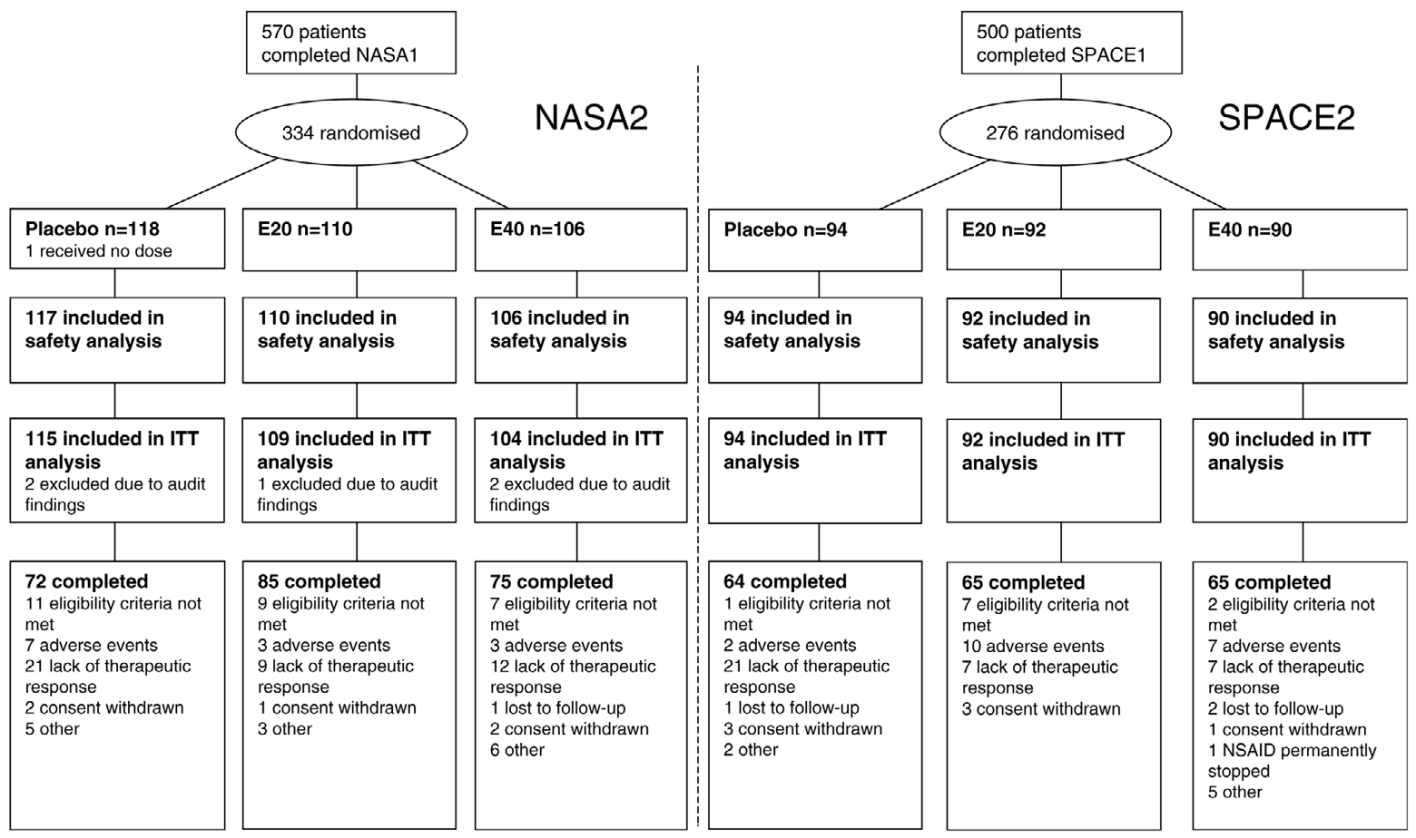
Flow diagram of patients through the studies. E20, esomeprazole 20 mg; E40, esomeprazole 40 mg; ITT, intention-to-treat; NSAID, non-steroidal anti-inflammatory drug.

Of 334 and 276 patients randomly assigned, 328 and 276 patients constituted the intention-to-treat populations for NASA2 and SPACE2, respectively. Enrolment for the NASA2 and SPACE2 studies started in February and April 2001, respectively, and the last patient completed both studies in February 2003. The flow of patients through the studies is shown in Figure 1. At study entry, the treatment groups were similar with respect to demographic and clinical characteristics, although slightly more patients receiving placebo during the maintenance phase had received esomeprazole 20 mg than either esomeprazole 40 mg or placebo during the acute phase (Table 1). The most common NSAIDs during maintenance treatment were rofecoxib (21%), celecoxib (20%), diclofenac (20%), ibuprofen (9%), piroxicam (4%), and naproxen (3%). The proportion of patients compliant with study drug (mean dosing: 75% to 125% relative to protocol) was more than 90% in all three treatment arms. Patient compliance with NSAID treatment (drugs used on at least 70% of days according to diary data) was similarly high (more than 90% of patients) in the three treatment arms in both studies. The diary card measurements demonstrated good reliability; the estimated correlation coefficient for the mean of 7 days of measurements (using GSRS abdominal pain score) was 0.62. Furthermore, the correlation between GSRS abdominal pain and the mean diary card measurements for the preceding 7 days ranged from 0.52 to 0.74 for the baseline values, last values, and change from baseline, indicating satisfactory validity.

### Efficacy

**Figure 2:**
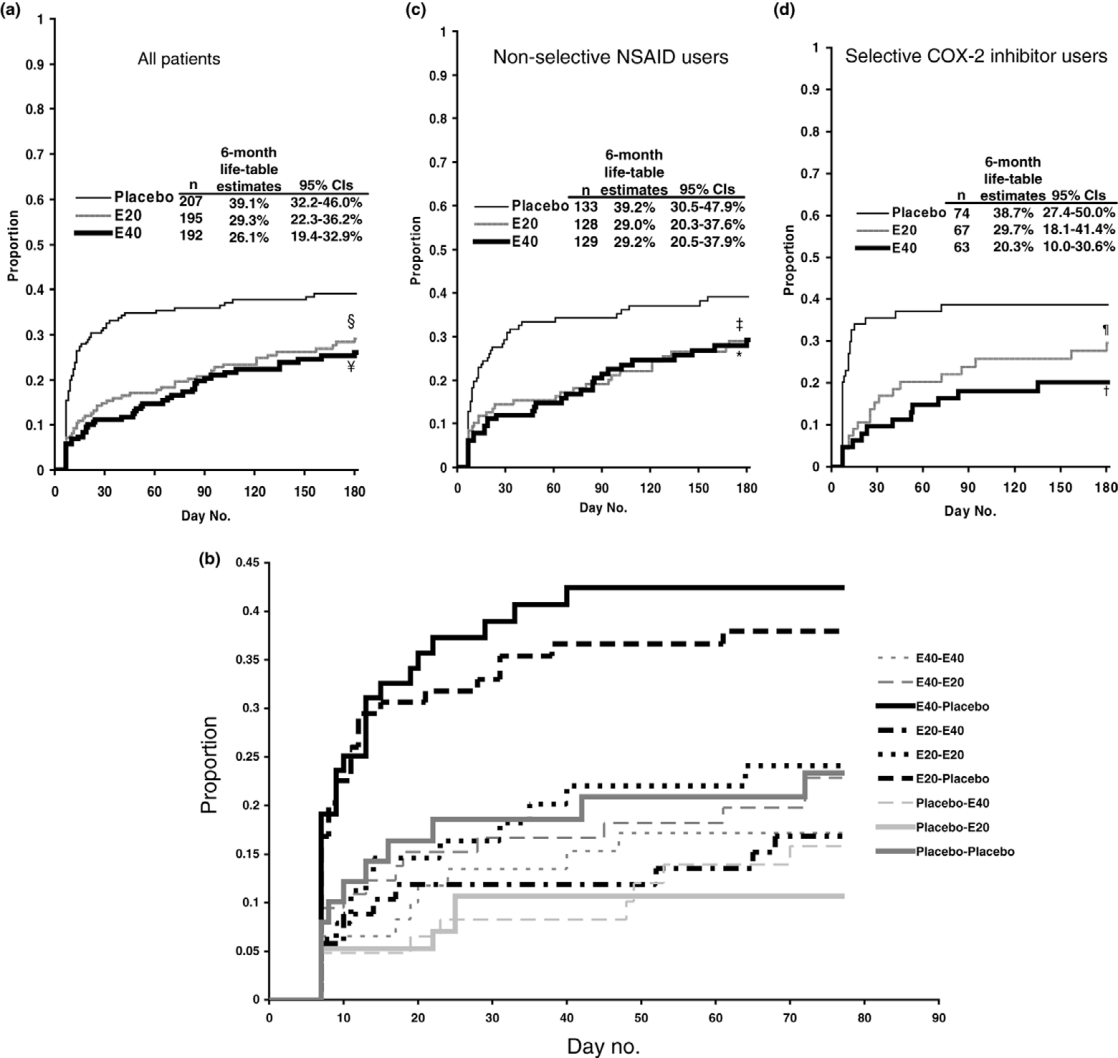
Estimated proportion of patients with relapse of upper gastrointestinal symptoms. Kaplan-Meier life-table analyses of the proportion of patients with relapse of pain, discomfort, or burning in the upper abdomen throughout 6 months of treatment with esomeprazole 20 mg (E20), esomeprazole 40 mg (E40), or placebo **(a) **for all patients, **(b) **according to treatment in the acute and maintenance studies, **(c) **for non-selective non-steroidal anti-inflammatory drug (NSAID) users, and **(d) **for selective cyclo-oxygenase-2 (COX-2) NSAID users (pooled intention-to-treat population). Diary card data were not available for 10 patients (placebo, *n *= 2; esomeprazole 20 mg, *n *= 6; esomeprazole 40 mg, *n *= 2). ^§^*p *= 0.006, ^¥^*p *= 0.001, ^‡^*p *= 0.03, *p = 0.02, ^¶^*p *= 0.08, ^†^*p *= 0.01, all versus placebo. CI, confidence interval.

Significantly fewer patients on esomeprazole 20 or 40 mg, compared with placebo, experienced relapse of upper GI symptoms (upper abdominal pain, discomfort, or burning) based on diary data (Figure 2a). Among patients who were treated with esomeprazole (either 20 or 40 mg) during the acute phase and who went on to receive placebo during the maintenance phase, high rates of relapse were observed (Figure 2b). Patients with an apparent response to placebo treatment during the acute phase had a lower relapse rate when treated with placebo during the maintenance phase than those who received active treatment during the maintenance phase. Relapse rates were, in general, lower in those who received esomeprazole during the maintenance phase, regardless of initial therapy. Data for the individual trials are shown in Table 2. Number needed to treat analysis showed that, throughout 6 months of treatment, 11 and 8 patients need to be treated with esomeprazole 20 mg or esomeprazole 40 mg, respectively, in order to avoid 1 patient experiencing relapse of upper GI symptoms. The exploratory regression analysis identified three factors as having a significant effect on time to relapse of upper GI symptoms. Treatment with esomeprazole 20 mg or esomeprazole 40 mg was associated with a significant reduction in upper GI symptoms (*p *= 0.0035 and *p *= 0.0017, respectively). Patients younger than 65 years old were significantly more likely than those 65 years old or older to experience a relapse of upper GI symptoms (*p *= 0.0284). No other variables included in the analysis were identified as having a significant effect on time to relapse of symptoms.

### Analysis by NSAID type

When patients taking non-selective NSAIDs or selective COX-2 inhibitors were analysed separately, fewer patients on esomeprazole experienced relapse of upper GI symptoms compared with those on placebo (Figure 2c,d). *Post hoc *analyses showed statistically significant differences for both doses of esomeprazole versus placebo among those using non-selective NSAIDs and for esomeprazole 40 mg among those using selective COX-2 inhibitors.

### Other individual investigator-assessed symptoms

**Figure 3:**
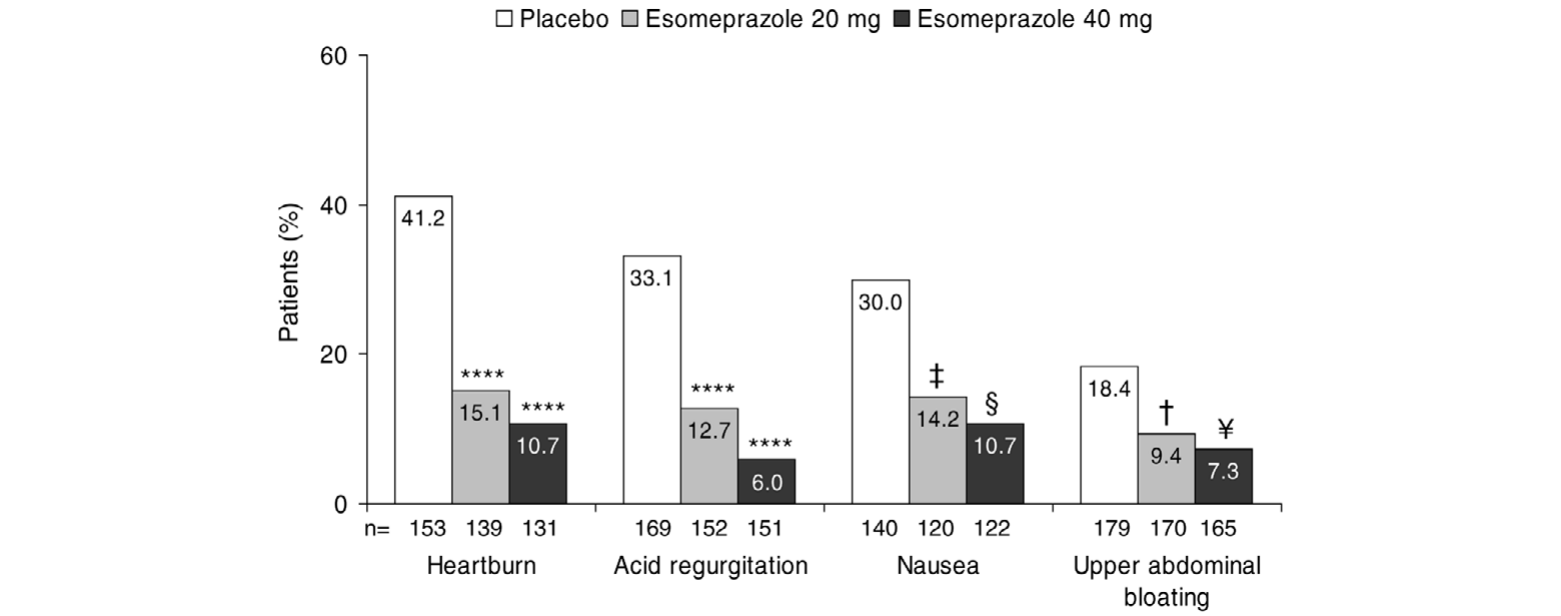
Proportion of patients with investigator-assessed symptoms in the week preceding the 6-month visit (pooled intention-to-treat population). Numbers (n) relate to patients without the individual symptom upon entering the 6-month maintenance phase. ^†^*p *= 0.02, ^¥^*p *= 0.002, ^‡^*p *= 0.003, ^§^*p *= 0.0001, ****p < 0.0001, all versus placebo.

As for upper abdominal pain, discomfort, or burning, the proportion of patients with investigator-assessed heartburn, acid regurgitation, nausea, and upper abdominal bloating at the 6-month visit was significantly lower in patients treated with esomeprazole (both 20 and 40 mg) than with placebo (Figure 3).

### Patient-reported outcomes

**Figure 4:**
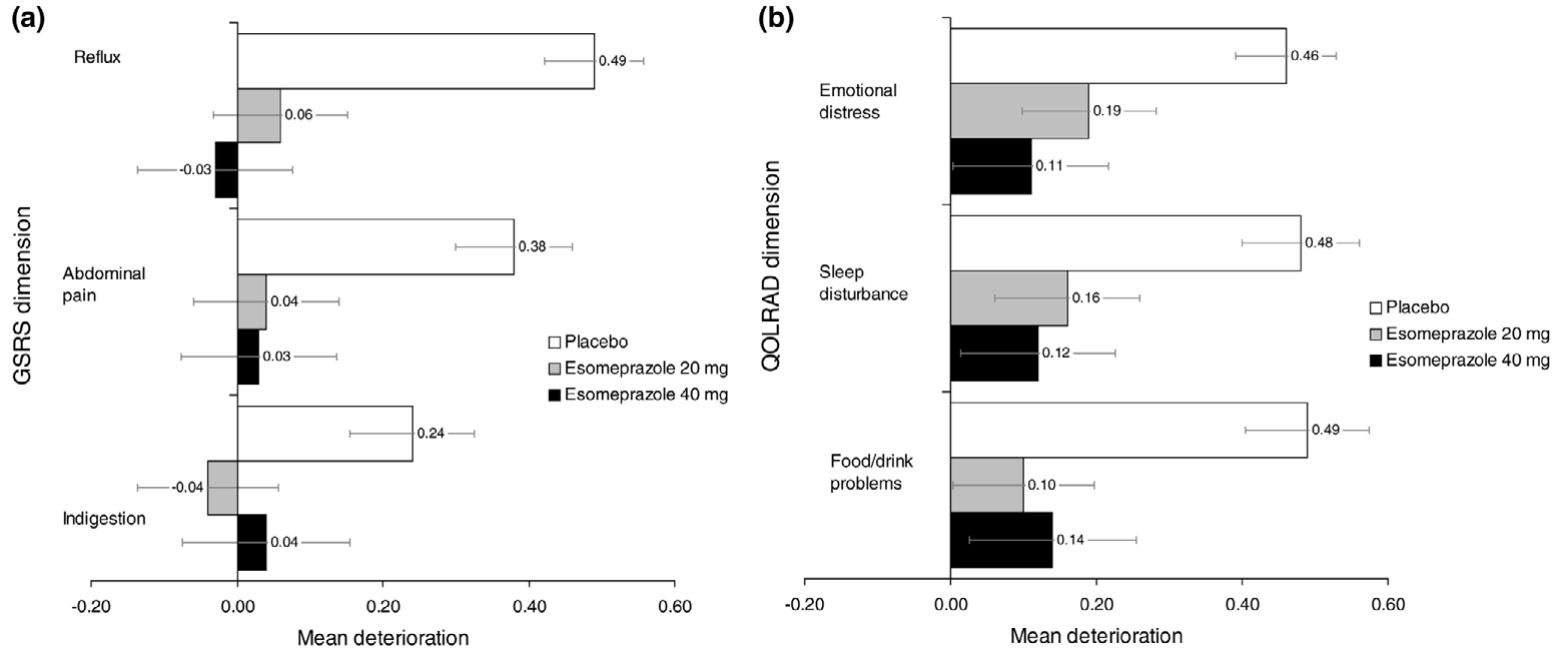
Score changes in patient-reported outcome scales. Mean (and standard error) score change in **(a) **Gastrointestinal Symptom Rating Scale (GSRS) and **(b) **Quality of Life in Reflux and Dyspepsia (QOLRAD) questionnaire (pooled intention-to-treat population).

Baseline GSRS mean scores for the three relevant dimensions were between 1.49 and 2.00 (1 = no symptoms, 7 = most severe symptoms). At 6 months, esomeprazole 20 mg and 40 mg were significantly more effective than placebo at maintaining previous symptom improvements for the GSRS dimensions of reflux (esomeprazole 20 mg: *p *= 0.002, esomeprazole 40 mg: *p *= 0.0002) and abdominal pain (esomeprazole 20 mg: *p *= 0.006, esomeprazole 40 mg: *p *= 0.002). A significant difference was also observed between esomeprazole 20 mg and placebo for the indigestion dimension (esomeprazole 20 mg: *p *= 0.03, esomeprazole 40 mg: *p *= 0.11) (Figure 4a).

Baseline QOLRAD mean scores for the three pre-specified relevant dimensions were between 6.30 and 6.59 (7 = no impairment of HRQL, 1 = most severe impairment) following improvements in the acute treatment studies [12]. At 6 months, esomeprazole 20 and 40 mg were significantly more effective than placebo for maintaining improvements in HRQL as judged by mean changes in the QOLRAD questionnaire dimensions of sleep disturbance (esomeprazole 20 mg: *p *= 0.02, esomeprazole 40 mg: *p *= 0.005) and food/drink problems (esomeprazole 20 mg: *p *= 0.003, esomeprazole 40 mg: *p *= 0.004). Esomeprazole 40 mg was also significantly more effective than placebo for the emotional distress dimension (esomeprazole 20 mg: *p *= 0.05, esomeprazole 40 mg: *p *= 0.004) (Figure 4b).

### Safety and tolerability

Esomeprazole 20 and 40 mg were well tolerated in combination with continuous daily NSAID use, and no safety concerns were raised. In the pooled population, AEs and discontinuations due to AEs occurred in 47.4% and 8.5% of placebo recipients, 55.9% and 7.4% of patients treated with esomeprazole 20 mg, and 54.6% and 6.1% of patients taking esomeprazole 40 mg, respectively. Serious AEs occurred in 5.2% of placebo recipients, 5.0% of patients taking esomeprazole 20 mg, and 7.1% of patients taking esomeprazole 40 mg. Neither of the two deaths (both due to pancreatic carcinoma, one in the placebo arm and one in the esomeprazole 40 mg arm) in the study was assessed as related to the study drug.

## Discussion

In the two 4-week studies that directly preceded the two studies reported here, esomeprazole 20 and 40 mg were shown to provide initial relief of NSAID-associated upper GI symptoms (upper abdominal pain, discomfort, or burning) [12]. The results presented here demonstrate that treatment with esomeprazole 20 and 40 mg maintained relief of these symptoms during a further 6 months of once-daily treatment. Esomeprazole-treated patients were also more likely to experience prolonged relief of other individual investigator-assessed upper GI symptoms than those taking placebo. Moreover, compared with those receiving placebo, esomeprazole-treated patients reported better GI symptom relief and HRQL as assessed using the GSRS and QOLRAD instruments.

The study had a number of limitations. First, patients were re-randomised from the acute studies even if their initial response had been to placebo, and this may have impacted on the study findings. For example, it appeared that patients taking placebo during the acute studies had a low relapse rate even when taking placebo during the maintenance phase; this is consistent with a true placebo response. In theory, the higher relapse rates in patients initially treated with esomeprazole who then received placebo during the maintenance phase could be attributed to acid rebound. However, it is difficult to distinguish between acid rebound and recurrence of initial symptoms, the latter of which seems more likely. Most of the benefits of acid suppression were established by day 45 (because most cases of symptom relapse in placebo recipients had already occurred by this time point). It is unknown whether those taking esomeprazole during the maintenance phase would require continued therapy beyond the 6-month time point in order to achieve sustained benefits. However, taking the two phases of this study as a whole, it seems likely that a substantial proportion of patients would experience relapse of symptoms if treatment were stopped at this point. In practice, prescribers may well assess the need for continued maintenance treatment by a trial of drug cessation.

In the two studies described here and the two 4-week studies that preceded them, NSAID-associated upper GI symptoms were strictly defined so as to differentiate them from symptoms more characteristic of GERD, primarily heartburn. For this reason, patients with a history of GERD were excluded from the studies. Moreover, the symptoms assessed as the primary variable in the study, namely 'upper abdominal pain, discomfort, or burning,' have been shown to be significantly increased by NSAID use [2].

Although selective COX-2 inhibitors appear to cause fewer upper GI events than NSAIDs, it is clear that drug-related upper GI symptoms remain a problem with this group of drugs [5,16]. When the studies were started, the use of selective COX-2 inhibitors was already substantial, and because upper GI symptoms requiring co-therapy are problematic for patients taking these drugs as well as those taking non-selective NSAIDs, our study design allowed trial entry on either type of drug. Similar reductions in upper GI symptom relapse rates to those seen in the whole study population were also observed for the subgroups, although esomeprazole 20 mg did not achieve statistical significance over placebo within the smaller selective COX-2 inhibitor group. These data, in conjunction with studies demonstrating that at-risk patients have a significant ulcer risk reduction with esomeprazole therapy [17], offer clinicians important treatment options for users of non-selective NSAIDs or selective COX-2 inhibitors. Our study also supports the previously established finding that dyspepsia is less prevalent in older patients than in younger patients [18,19]. However, whether this is due to a true reduction, reduced perception, or a disinclination to report symptoms is unknown.

Limited data are available on long-term symptom control in patients with dyspepsia. Our results compare favourably with those of one randomised controlled trial in *H. pylori*-negative patients with dyspepsia [20]. Patients received omeprazole 20 mg once daily, ranitidine 150 mg once daily, cisapride 20 mg twice daily, or placebo for 4 weeks followed by 5 months of on-demand treatment with the same therapy. The proportions of patients with symptom relapse (that is, score of more than or equal to 3 on an amended 7-point Likert scale) at 6 months were 56% for omeprazole, 59% for ranitidine, 60% for cisapride, and 65% for placebo [20]. In our study, relapse rates of 26.1% to 39.1% were observed. However, due to differences in study design between this study and our study, it is difficult to draw conclusions from these data.

## Conclusion

The future use of selective COX-2 inhibitors is uncertain following the withdrawal of rofecoxib due to the risk of cardiovascular events. The results of these studies, however, show a benefit with esomeprazole therapy (20 and 40 mg) among patients taking either non-selective NSAIDs or selective COX-2 inhibitors. These findings are of particular relevance for patients requiring long-term, continuous anti-inflammatory therapy, who may otherwise need to discontinue treatment because of upper GI side effects.

## Abbreviations

AE = adverse event; COX-2 = cyclo-oxygenase-2; GERD = gastroesophageal reflux disease; GI = gastrointestinal; GSRS = Gastrointestinal Symptom Rating Scale; HRQL = health-related quality of life; NASA = Nexium Anti-inflammatory Symptom Amelioration; NSAID = non-steroidal anti-inflammatory drug; PPI = proton pump inhibitor; QOLRAD = Quality of Life in Reflux and Dyspepsia; SPACE = Symptom Prevention by Acid Control with Esomeprazole.

## Competing interests

CJH has received research funding and/or honoraria from AstraZeneca (Mölndal, Sweden), GlaxoSmithKline (Uxbridge, Middlesex, UK), Merck (Whitehouse Station, NJ, USA), NitroMed, Inc. (Lexington, MA, USA), Novartis International AG (Basel, Switzerland), Pfizer, Inc. (New York, NY, USA), Takeda Pharmaceutical Company Limited (Osaka, Japan), Serono International SA (Geneva, Switzerland), Wyeth (Madison, NJ, USA), and Grünenthal GmbH (Aachen, Germany). NJT has participated in consultant advisory boards sponsored by AstraZeneca and has received consultant's research support from TAP Pharmaceutical Products, Inc. (Lake Forest, IL, USA). JMS has received grant/research support from AstraZeneca, is a consultant to AstraZeneca, Merck, Novartis International AG, TAP Pharmaceutical Products, Inc., Pfizer, Inc., The GI Company, Inc. (Framingham, MA, USA), POZEN Inc. (Chapel Hill, NC, USA), Bayer Corp. (Emeryville, CA, USA), and GlaxoSmithKline, and has participated in Speaker's Bureaus for AstraZeneca, TAP Pharmaceutical Products, Inc., and Santarus, Inc. (San Diego, CA, USA). RHJ has received consultancy and speaker fees from AstraZeneca, Altana AG (Bad Homburg, Germany), and Reckitt Benckiser plc (Slough, Berkshire, UK). GL and JN are full-time employees of AstraZeneca. NDY has received speaker fees from a maker of PPIs, AstraZeneca, which financed the development of this manuscript.

## Authors' contributions

All authors were involved in study design and manuscript preparation. CJH, NJT, JMS, RHJ, and NDY were responsible for data acquisition and interpretation. JN was involved in data interpretation. Data analysis was provided by GL. All authors read and approved the final manuscript.

## Figures and Tables

**Table 1:** Baseline demographic and clinical characteristics of the pooled study populations

	Pooled population		
	
Characteristic	Placebo (*n *= 209)	Esomeprazole 20 mg (*n *= 201)	Esomeprazole 40 mg (*n *= 194)
Male/female gender, percentage	27.3:72.7	21.4:78.6	24.2: 75.8
Age in years, mean (standard deviation)	57.1 (13.4)	54.4 (11.9)	55.7 (13.6)
Type of chronic condition, percentage			
Rheumatoid arthritis	24.4	18.4	20.6
Osteoarthritis	43.1	45.3	42.8
Other chronic condition	32.5	36.3	36.6
Time since first diagnosis of chronic condition in years, median	9	7	9
*Helicobacter pylori *status (histology), percentage^a^			
Negative	95.7	93.0	95.9
Positive	3.3	6.5	3.1
Unknown	1.0	0.5	1.0
NSAID type, percentage			
Non-selective	64.1	65.7	67.0
Selective COX-2 inhibitor	35.9	34.3	33.0
Study drug in acute study, percentage			
Placebo	24.4	29.3	32.5
Esomeprazole 20 mg	43.1	32.8	36.1
Esomeprazole 40 mg	32.5	37.8	31.4

^a^*H. pylori *status derived from the acute studies (NASA1 [Nexium Anti-inflammatory Symptom Amelioration] and SPACE1 [Symptom Prevention by Acid Control with Esomeprazole]). COX-2, cyclo-oxygenase-2; NSAID, non-steroidal anti-inflammatory drug.

**Table 2:** Estimated proportion of patients with relapse of upper gastrointestinal symptoms

	NASA2 study	*p *value versus placebo	SPACE2 study	*p *value versus placebo
Placebo	38.2%	-	39.8%	-
Esomeprazole 20 mg	28.3%	0.04	30.7%	0.07
Esomeprazole 40 mg	24.3%	0.004	28.4%	0.05

Kaplan-Meier life-table estimates of proportion of patients with relapse of pain, discomfort, or burning in the upper abdomen throughout 6 months of treatment with esomeprazole 20 mg, esomeprazole 40 mg, or placebo and log-rank comparisons for the individual studies. NASA, Nexium Anti-inflammatory Symptom Amelioration; SPACE, Symptom Prevention by Acid Control with Esomeprazole.
